# Supplementary material for: Clinical characteristics, comorbidities, and correlation with advanced lipedema stages: A retrospective study from a Swiss referral centre
Source: PLoS One. 2025 Mar 20;20(3):e0319099. doi: 10.1371/journal.pone.0319099 (PMC11925301; doi:10.1371/journal.pone.0319099)
Supplement: S1 Table — (DOCX) [file pone.0319099.s001.docx]

| **Supplementary Table 1.** Symptoms across lipedema stages | | | | |
| --- | --- | --- | --- | --- |
|  | **Stage 1** | **Stage 2** | **Stage 3-4** | **Total** |
|  | (N=101) | (N=169) | (N=110) | (N=380) |
| Easy bruising | 81 (80.2%) | 129 (76.3%) | 85 (77.3%) | 295 (77.6%) |
| Pain | 89 (88.1%) | 147 (87.0%) | 98 (89.1%) | 334 (87.9%) |
| Heaviness | 83 (82.2%) | 125 (74.0%) | 80 (72.7%) | 288 (75.8%) |
| Disproportionate enlargement of limbs | 82 (81.2%) | 132 (78.1%) | 99 (90.0%) | 313 (82.4%) |
| Stemmer positive | 4 (4.0%) | 5 (3.0%) | 6 (5.5%) | 15 (3.9%) |
| Hypermobility | 14 (13.9%) | 11 (6.5%) | 4 (3.6%) | 29 (7.6%) |
| a) Hypermobility was classified into two groups: non-hypermobile (score <4) and hypermobile (score ≥4) | | | | |
